# Impact of Umbilical Cord Blood-Derived Mesenchymal Stem Cells on Cardiovascular Research

**DOI:** 10.1155/2015/975302

**Published:** 2015-03-10

**Authors:** Santiago Roura, Josep Maria Pujal, Carolina Gálvez-Montón, Antoni Bayes-Genis

**Affiliations:** ^1^ICREC Research Program, Germans Trias i Pujol Health Science Research Institute, Can Ruti Campus, 08916 Badalona, Spain; ^2^Cell Processing Laboratory, Parc Científic i Tecnològic Universitat de Girona, 17003 Girona, Spain; ^3^Cardiology Service, Germans Trias i Pujol University Hospital, 08916 Badalona, Spain; ^4^Department of Medicine, UAB, Barcelona, Spain

## Abstract

Over the years, cell therapy has become an exciting opportunity to treat human diseases. Early enthusiasm using adult stem cell sources has been tempered in light of preliminary benefits in patients. Considerable efforts have been dedicated, therefore, to explore alternative cells such as those extracted from umbilical cord blood (UCB). In line, UCB banking has become a popular possibility to preserve potentially life-saving cells that are usually discarded after birth, and the number of UCB banks has grown worldwide. Thus, a brief overview on the categories of UCB banks as well as the properties, challenges, and impact of UCB-derived mesenchymal stem cells (MSCs) on the area of cardiovascular research is presented. Taken together, the experience recounted here shows that UCBMSCs are envisioned as attractive therapeutic candidates against human disorders arising and/or progressing with vascular deficit.

## 1. Introduction

Over the years, cell therapy has become an exciting opportunity to treat human diseases [1–4]. In line, considerable preclinical efforts have been dedicated to exploring valuable stem cell sources, including those from umbilical cord blood (UCB). In this context, UCB extraction as well as banking is a way to preserve potentially life-saving cells that are usually discarded after birth and has become a popular possibility among expectant parents thinking about promising options to secure their child's life. But what benefit is associated with the long and high-cost procedure that is necessary to isolate and store cells for 25 to 30 years? Public banks offer the option of altruistic donation, whereas in private banks cellular products are conserved for own use. Hybrid models blending aspects of both public and private banking are also currently intended. However, there are questions regarding the cost* versus* the benefits of UCB banking, and it also raises complex ethical and legal issues. Given the variety of existing options and familial and personal interests, there is a definite need for careful regulation of UCB banking and applications [5–7].

In the following pages of this review we recount some of the most relevant issues regarding categories of UCB processing laboratories and banks, as well as the properties, challenges, and impact of UCB-derived mesenchymal stem cells (MSCs) on the area of cardiovascular research.

## 2. Categories of UCB Banks

The processing of large numbers of UCB units is partially automated. In brief, once UCB samples arrive at processing laboratory, a cell suspension enriched with mononuclear cells—where the stem cell population resides in—is collected following sedimentation of red blood cells and centrifugation under high sterile conditions. The resultant cell product is cryopreserved following a controlled rate freezing process to slowly reduce the temperature to −180°C and stored in liquid nitrogen dewars [8, 9]. Alternatively, for subsequent isolation and expansion of mesenchymal-like stem cell colonies, 30% fetal calf serum, low-glucose DMEM medium supplemented with 10^−7^ M dexamethasone, and closed system applying cell stacks are used. The resultant cell product can also be stored frozen, thawed, and expanded further in clinical grade quality [10, 11].

In order to appropriately preserve donated units for human therapies, a number of UCB banks have been created worldwide [6]. Originally, these laboratories were run by hospitals or nonprofit institutions, which processed the donated samples and provided cells to patients when needed. Accredited “public” UCB banks were subsequently linked to national registries, which in turn were linked to international inventories. This coordination has favored the identification of the most suitable sample for each patient who requires a transplant [6]. More recently, because private companies have been offering UCB storage for own use or for the use of close relatives, UCB banks can be classified into the following categories: private or public and for-profit or nonprofit. By definition, public (nonprofit) banks store UCB-derived cells and provide them when transplantation is prescribed to patients without regard for filial relationships, while private firms offer a commercial service to parents to preserve UCB-derived cells for expected progeny. However, alternatives to private banks have recently emerged. These include mixed or hybrid private-public banks, such as that proposed by the Virgin Health Bank; in the Spanish system, autologous samples can be stored and are given to individuals other than the donor if required [6, 7].

## 3. UCBMSCs: Properties and Challenges

UCB is currently considered the most plentiful stem cell reservoir for clinical applications [12, 13]. Although used mainly for hematopoietic progenitor cell (HPC) transplantation against blood disorders, the spectrum of diseases for which UCB provides effective therapy has been expanded to include nonhematopoietic conditions, including cell-based regenerative therapy and immune modulation. This undeniable fact is being reinforced because, as mentioned above, UCB also contains MSCs. MSCs were sought to be present in UCB at a low frequency in contrast to their presence in other tissues throughout the body, including bone marrow, adipose tissue, placenta, dermis, and umbilical cord [14–19]. However, transplantation of double partially HLA-matched UCB units is recognized as a simple approach for overcoming this marked limitation [12, 13]. Remarkably, recent work shows that MSCs can also be expanded successfully from 30% to 60% of low-volume UCB units [20]. In terms of advantages, UCB can be safely and painlessly extracted and long-term cryopreserved and has a lower risk of transmitting viral infections or somatic mutations than adult tissues (i.e., bone marrow).

Commonly, MSCs are recognized by their capability to differentiate into osteogenic, chondrogenic, and adipogenic lineages* in vitro*, typical mesenchymal-like morphology, adherence to plastic when maintained in standard culture conditions, and nonhematopoietic cell surface pattern according to the International Society of Cellular Therapy (ISCT) criteria [21]. Distinct populations of mesenchymal-like stem cells with similar adhesion properties and antigen surface expression patterns but different pluripotency have been isolated from UCB. In brief, Kögler et al. described intrinsically pluripotent or unrestricted somatic cells with the potential to reprogram into mesodermal, endodermal, and ectodermal fates [22]. Subsequently, other investigators used MSCs from UCB with more restricted pluripotency [23]. These “conflicting” data show the great cell heterogeneity in terms of growth and differentiation potential that has a major impact on the envisioned therapeutic application of MSCs, including those from UCB [24]. Since the ISCT-proposed criteria were published in 2006, some advances have been performed in an attempt to decrease substantial ambiguities in MSC definition and verification. As a result, additional cell surface markers such as STRO-1, CD271, CD200, Ganglioside GD2, Frizzled-9, and tissue nonspecific alkaline phosphatase have been included to verify identity of the isolated cells. Furthermore, together with specific staining after trilineage differentiation, the use of commercially available functional kits provides a systematic verification of MSC identity, independently of the tissue or species type.

In the context of cell-based therapies, due to their great proliferative activity, less culture time is required to get a fixed number of* ex vivo* expanded UCBMSCs and, therefore, fewer chances to apoptotic features. Taking into account that MSCs possess the greatest degree of multipotency, there is a need to standardize MSC isolation and culture procedures. For example, enzymatic or explant-based methods may not lead to the same cell types [25–28]. In addition, although human MSCs do not undergo malignant transformation during* in vitro* expansion [29, 30], they experience replicative senescence and mutational acquisition [30, 31]. Remarkably, Bellayr et al. have identified specific markers that distinguish aging bone marrow MSCs grown in cell culture [32]. These authors argue that confirmatory studies are needed to develop specific assays to test the quality of MSCs before any clinical use. Other options could be based on tissue transposition directly to regeneration-desired sites without stem cell extraction and long-term culture.

Moreover, solid organ transplantation is the unique solution for end-stage organ failure. Only in 2012, it is estimated that about 115,000 solid organ transplants were performed worldwide [33]. However, alternative treatments to the chronic use of immunosuppressive drugs in order to avoid rejection episodes conducted by the recipient's immune system and increase donor-specific tolerance are under investigation. They include different types of regulatory cells, for example, MSCs which have been evaluated with promising results [34]. In terms of immunogenicity, UCBMSCs have inherent “immunoprivileged” properties. UCBMSCs are characterized to express class I human leukocyte antigens (HLA antigens), whereas class II HLA antigens are expressed only after sustained exposure to interferon-*γ* [35–38]. The lower immunogenicity of UCBMSCs is attributed to its immaturity, in contrast to alternative adult stem cell sources. Accordingly, UCBMSCs may be used for allogeneic transplantation [39]. However, recent findings on MSCs inducing a systemic inflammatory response within hours after infusion [40] need to be tested using those from UCB and likely solved for the application of these cells into the clinic.

## 4. UCBMSCs and Cardiovascular Diseases: An Active Area of Research

Since MSCs were reported to differentiate* in vitro* into a myogenic phenotype, the benefit of treating ischemic and nonischemic cardiovascular disorders with these cells (mainly those extracted from bone marrow) has been demonstrated and supported with compelling evidence [41]. Briefly, paramount milestones in this field include the differentiation of bone marrow-derived MSCs into cardiomyocyte-like cells* in vitro* [42]; the implantation of autologous MSCs cultured from bone marrow into a rat heart at 3 weeks after cryoinjury [43]; a report that described progress after 10 years of cell-based cardiac repair [44]; and, most recently, the Cardiopoietic stem Cell therapy in heart failURE (C-CURE) clinical trial based on the use of autologous bone marrow-derived and cardiogenically oriented MSCs in patients with chronic heart failure [45].

**Figure 1:**
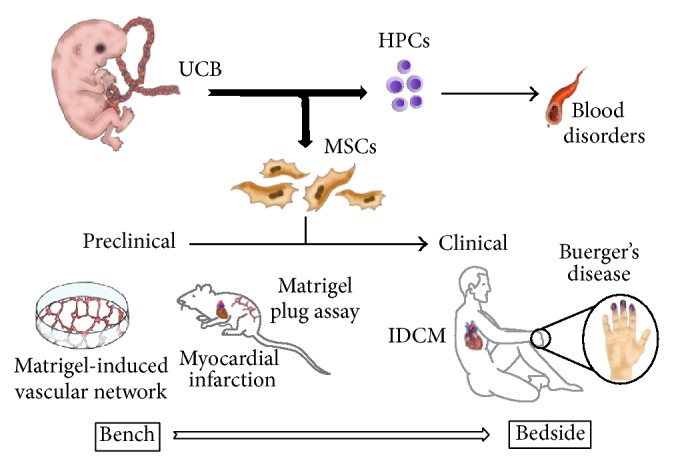
Use of UCBMSCs in the field of cardiovascular research. Scheme summarizing the main preclinical and clinical settings in which UCBMSCs are being employed. In brief, since the first transplant in 1988, UCB has increasingly been employed as an alternative source of HPCs for transplantation against human blood diseases. UCB also contains MSCs which have garnered a great deal of attention to treat cardiovascular diseases such as idiopathic dilated cardiomyopathy and Buerger's disease. Additionally, considerable preclinical efforts have been directed to explore basic UCBMSC properties and molecular mechanisms* in vitro*, as well as their behavior and functions once implanted* in vivo*. UCB: umbilical cord blood; HPC: hematopoietic progenitor cell; MSC: mesenchymal stem cell; IDCM: idiopathic dilated cardiomyopathy. Designed and hand-drawn by Carolina Gálvez-Montón.

In addition, UCBMSCs are actively being used in the cardiovascular area of study. The most relevant preclinical and clinical studies using these cells are discussed below. In line, some of the experiences reported in this field of research are collectively summarized in Figure 1.

### 4.1. Preclinical Studies

In the preclinical setting, for instance, Erices et al. investigated the homing capacity of transplanted human UCBMSCs in the bone marrow of unconditioned nude mice. As a result, after systemic infusion, they also found human DNA in cardiac muscle, as well as in other recipient tissues [46]. Since then, one central unanswered question has been whether these cells have cardiomyogenic potential. Although several investigators have reported the differentiation of UCBMSCs into the cardiomyogenic lineage* in vitro* [47, 48], others have failed to demonstrate such differentiation [49, 50] using a broad range of procardiogenic stimuli, including 5-azacytidine [48], dimethyl sulfoxide [49], a combination of growth factors involved in early cardiomyogenesis [50], activation of Wnt signaling pathways [51, 52], and coculture with neonatal rat cardiomyocytes [53]. A coculture system using rat cardiomyocytes had been effective in inducing a cardiomyocyte-like phenotype in CD133^+^ hematopoietic progenitors that were first selected from UCB by immunomagnetic separation and then expanded by stimulation with platelet-derived and epidermal growth factors [54]. When cultured for up to 4 weeks in a cardiac differentiation-promoting medium, this same cell population gained the expression of a variety of paramount cardiac-specific proteins [55]. However, other authors did not find that direct contact with neonatal rat cardiomyocytes promoted either the expression of cardiomyocyte-specific proteins, or the presence of rhythmic calcium oscillations and potential-dependent fluorescence emission in UCBMSCs [50]. Thus, they concluded that alternative strategies, regulatory factors, or signaling clues might be better suited to recruit UCBMSCs into the cardiac cell lineage. Surprisingly, a nonhematopoietic cellular subpopulation within the mononuclear cell fraction isolated from UCB was differentiated towards the cardiomyogenic lineage after these cells were cocultured with brown adipose tissue-derived cells [56]. Further details about the cardiomyogenic potential of UCBMSCs from ongoing investigations will have to shine light on this essential question and allow the design of effective cardiac cell therapy using these cells.

In recent times, several authors have suggested that MSCs may also play a role in vascular growth, garnering a great deal of attention for therapeutic purposes [14]. In line, intramyocardially administered CD105^+^ UCBMSCs exhibited favorable survival in infarcted mouse hearts, which translates into better capillary density in both border and remote zones 6 weeks after infarction and more robust preservation of cardiac function [57]. Lee et al. also found that N-cadherin determines individual variations in the therapeutic efficacy of human UCBMSCs in a rat myocardial infarction model and that variations in capillary density are correlated with therapeutic efficacy in improving left ventricular function [58].

Currently, noninvasive techniques such as bioluminescence imaging, which is based on the application of natural reactants with light-emitting capabilities (photoproteins and luciferases), provide valuable information about cardiac cell transplantation in living animals [59]. Indeed, combination of this advanced nondestructive imaging technique with reporter gene technology has been useful to improve outcomes of cell therapies in the field of cardiac regeneration. This is, for instance, the case of the monitoring of chimeric luciferase/fluorescent protein expression by human engineered UCBMSCs in a mouse model of angiogenesis (Matrigel Plug Assay) [60]. In particular, an efficient differentiation of these genetically modified cells into the endothelial cell lineage was demonstrated. The implanted cells also self-organized into new functional blood vessels connected with the host circulatory system; these newly formed vascular structures were filled with high molecular weight FITC-dextran injected through the lateral tail vein of mice before sacrifice. In the same study, engineered UCBMSCs were mixed with fibrin and applied as an adhesive patch over infarcted myocardium wound to analyze their potential benefits. As a result, the implanted cells proliferated early, survived during 4 weeks over injured myocardium, efficiently differentiated towards the endothelial lineage, and induced the development of new functional vasculature. Furthermore, although no cells were found migrating from the patch to the myocardium of infarcted animals, they formed vascular-like structures expressing CD31 (a surface endothelial cell-specific antigen). Importantly, cell-treated animals also exhibited reduced infarct size and larger vessel than controls. However, it remains to be elucidated whether the implantation of this newly designed bioprosthesis promotes a significant general recovery of lost myocardial functions after myocardial infarction.

### 4.2. Clinical Studies

In the clinical setting, there are UCB-based treatments for both hematological and nonhematological conditions, including neurological diseases, diabetes mellitus, hepatic/gastrointestinal alterations, skin diseases, rheumatoid arthritis, systemic lupus erythematosus, bronchopulmonary dysplasia, malignant solid tumors, hematologic malignancies, inborn metabolic disorders, orthopedic cartilage repair, and osteopetrosis [61, 62]. In particular, neurological disorders represent the most commonly published area of expertise and the most active area of study in ongoing registered trials. Furthermore distinct cell types (total nucleated cells, mononuclear cells, and CD34-selected HPCs) and delivery routes (intravenous, intrathecal, subcutaneous, and intramuscular) are used [61]. To date, although being an active field of research, UCBMSCs have been less tested clinically. In the field of cardiovascular diseases, Kim and coworkers transplanted human leukocyte antigen-matched UCBMSCs into four men with Buerger's disease—a nonatherosclerotic, inflammatory, vasoocclusive disorder—who had already received medical treatment and surgical therapies [63]. After transplantation procedure, ischemic rest pain suddenly disappeared from their affected extremities and, in the follow-up angiography, digital capillaries were increased in number and size. In addition, vascular resistance in the affected extremities, compared with the preoperative examination, was markedly decreased due to improvement of the peripheral circulation. The authors concluded that implanted cells could incorporate to arterial walls of the ischemic hind limb in the treated group and suggested that the use of human UCBMSCs could be a new and useful therapeutic weapon for ischemic diseases. Moreover, we have recently showed that UCBMSCs are conceivably new therapeutic agents for patients afflicted by idiopathic dilated cardiomyopathy, in which disease progression has the signature of marked cardiac endothelial deficiencies [64].

## 5. Conclusions

Together with somatic reprogramming induced by gene transfer [65], cell therapy has been an exciting innovation to treat human diseases. However, early enthusiasm using adult stem cell sources has been tempered in light of preliminary benefits in patients. Considerable efforts have been dedicated, therefore, to explore alternative cells such as those extracted from UCB. Since the first transplant in 1988, UCB has increasingly been employed for transplantation of hematopoietic cells in blood diseases and the number of UCB banks has grown worldwide [66]. In particular, public banks offer the option of altruistic donation, whereas in private banks cellular products are conserved for own use. Hybrid models blending aspects of both public and private banking are currently planned [67]. In this context, taking into consideration the experience recounted here, UCBMSCs are also envisioned as attractive therapeutic candidates against human disorders, for example, those arising and/or progressing mainly by vascular deficits.
